# Acupuncture Stimulation Alleviates Corticosterone-Induced Impairments of Spatial Memory and Cholinergic Neurons in Rats

**DOI:** 10.1155/2012/670536

**Published:** 2011-12-15

**Authors:** Bombi Lee, Bong-Jun Sur, Sunoh Kwon, Euntaek Jung, Insop Shim, Hyejung Lee, Dae-Hyun Hahm

**Affiliations:** ^1^Acupuncture and Meridian Science Research Center, College of Oriental Medicine, Kyung Hee University, 1 Hoegi-dong, Dongdaemun-gu, Seoul 130-701, Republic of Korea; ^2^Department of Oriental Medicine, Graduate School of Oriental Medicine, Kyung Hee University, 1 Hoegi-dong, Dongdaemun-gu, Seoul 130-701, Republic of Korea

## Abstract

The purpose of this study was to examine whether acupuncture improves spatial cognitive impairment induced by repeated corticosterone (CORT) administration in rats. The effect of acupuncture on the acetylcholinergic system was also investigated in the hippocampus. Male rats were subcutaneously injected with CORT (5 mg/kg) once daily for 21 days. Acupuncture stimulation was performed at the HT7 (Sinmun) acupoint for 5 min before CORT injection. HT7 acupoint is located at the end of transverse crease of ulnar wrist of forepaw. In CORT-treated rats, reduced spatial cognitive function was associated with significant increases in plasma CORT level (+36%) and hippocampal CORT level (+204%) compared with saline-treated rats. Acupuncture stimulation improved the escape latency for finding the platform in the Morris water maze. Consistently, the acupuncture significantly alleviated memory-associated decreases in cholinergic immunoreactivity and mRNA expression of BDNF and CREB in the hippocampus. These findings demonstrate that stimulation of HT7 acupoint produced significant neuroprotective activity against the neuronal impairment and memory dysfunction.

## 1. Introduction

Acupuncture has long been known to modulate the biochemical balances in the central nervous system (CNS) and to maintain homeostasis [1]. Recently, much attention has been paid to acupuncture as an alternative therapy to improve memory-deficit symptoms through modulating the hypothalamic-pituitary-adrenal (HPA) axis and chronic stress-induced neurobiological responses [2–4]. In behavioral studies using a rat stress model induced by immobilization or chronic injection of corticosterone (CORT), we have previously demonstrated that hand acupuncture and low-frequency electroacupuncture stimulation significantly improved depression-like and anxiety-like behavior and restored the expression levels of neuropeptide Y and c-Fos in the brain [3, 5]. However, the underlying mechanism of acupuncture stimulation in modulating spatial cognitive function with respect to the cholinergic system in the CNS remains poorly understood.

Chronic stress causes dysregulation of the HPA axis in the neuroendocrine system, as evidenced by observations that the elevation of circulating CORT levels disrupts circadian regulation of CORT secretion as well as the glucocorticoid (GC) receptor-negative feedback circuit [6, 7]. Whereas acute stress increases GC levels and stimulates cognitive function, thus facilitating memory consolidation [8], prolonged and repeated exposure to high GC levels evokes adverse effects on cognition and memory function in rodents and humans [9, 10]. Accordingly, adrenalectomy or the restoration of CORT to normal blood levels prevents stress-induced cognitive deficits [11]. Many studies have shown that stimulation and sustained action of the HPA axis is attenuated via negative feedback actions of circulating GC by exogenous CORT administration, and this is closely associated with the development of psychosomatic disorders and produces serious changes in affective behavior indicative of memory deficit symptoms [12, 13].

The mammalian hippocampus has the highest density of GC receptors and participates in GC-mediated negative feedback of the HPA axis [14]. Therefore, hippocampal neurons, which are known to play an important role in learning and memory function, are particularly vulnerable to neuronal injury induced by chronic CORT injection [15], resulting in deficits in spatial memory and synaptic plasticity [16, 17].

The aim of the present study was to explore the efficacy of acupuncture therapy for healing chronic CORT-induced spatial memory impairment in an animal model using behavioral and neurobiological methodologies. To this end, acupuncture stimulation of HT7 (Sinmun) was evaluated for its efficacy in alleviating spatial learning and memory deficits in rats repeatedly exposed to exogenous CORT using the Morris water maze (MWM) test, and its underlying mechanism was elucidated by analyzing cholinergic markers in the hippocampus.

## 2. Materials and Methods

### 2.1. Animals

Adult male Sprague-Dawley (SD) rats weighing 260–280 g were obtained from Sam taco Animal Co. (Seoul, Korea). The rats were housed in a limited-access rodent facility with up to five rats per polycarbonate cage. The room controls were set to maintain the temperature at 22°C ± 2°C and the relative humidity at 55% ± 15%. Cages were lit by artificial light for 12 h each day. Sterilized drinking water and standard chow diet were supplied ad libitum to each cage during the experiments. The animal experiments were conducted in accordance with the National Institutes of Health *Guide for the Care and Use of Laboratory Animals* (NIH Publications no. 80-23), revised in 1996 and were approved by the Kyung Hee University Institutional Animal Care and Use Committee. All animal experiments began at least 7 days after the animals arrived.

### 2.2. Experimental Groups

#### 2.2.1. Experiment 1

This study was designed to compare the CORT concentration in rat blood and brain tissue, immediately after CORT injection or immobilization stress for 21 days. The rats were randomly divided into three groups of four individuals each as follows: nontreated normal group (NOR group, *n* = 4), restraint-stressed group (STR group, *n* = 4), and CORT-injected and nontreated group (5 mg/kg, s.c., CORT group, *n* = 4). The immobilization stress procedure was performed by daily placing animals in 20 × 7-cm plastic tubes for 2 h for 21 days [18]. There were several 3-mm holes at one end of a tube for breathing. The animals had ample air but were unable to move.

Also, the other experiment was designed to compare the effects of acupuncture stimulation to enhanced CORT concentration in the blood of chronic CORT-injected rats for 21 days. The rats were randomly divided into five groups of four individuals each as follows: vehicle saline-injected group, instead of CORT (0.9% NaCl, s.c., CON group, *n* = 4), CORT-injected and nontreated group (5 mg/kg, s.c., CORT group; *n* = 4), CORT-injected and Sinmun (HT7) acupoint-stimulated group (CORT-HT group; *n* = 4), CORT-injected and Waiguan (TE5) acupoint-stimulated group (CORT-TE group; *n* = 4), CORT-injected and nonacupoint- (on the tail) stimulated group (CORT-TA group; *n* = 4). Corticosterone (Sigma-Aldrich Chemical Co., St Louis, mo, USA), dissolved in absolute ethanol, and subsequently diluted in water to the final concentration of 10% ethanol was injected at a daily dose of 5 mg/kg [19]. This CORT dose was selected because it induces plasma levels of the steroid comparable to those elicited by substantial stress. The CORT and vehicle injections were given in the morning between 9 and 10 am once daily for 21 consecutive days. As a vehicle control, animals in the CON group were subcutaneously given the equivalent volumes of saline to the final concentration of 10% ethanol. The daily doses and duration were determined based on the previous studies by Trofimiuk et al. [19].

#### 2.2.2. Experiment 2

This study was designed to explore the efficacy of acupuncture therapy for healing chronic CORT-induced spatial memory impairment in an animal model using behavioral and neurobiological methodologies. The rats were randomly assigned to divided into five groups of seven individuals each as follows; vehicle saline-injected group (CON group, *n* = 7), CORT-injected and nontreated group (CORT group; *n* = 7), CORT-injected and Sinmun (HT7) acupoint-stimulated group (CORT-HT group; *n* = 7), CORT-injected and Waiguan (TE5) acupoint-stimulated group (CORT-TE group; *n* = 7), CORT-injected and nonacupoint- (on the tail) stimulated group (CORT-TA group; *n* = 7).

**Figure 1:**
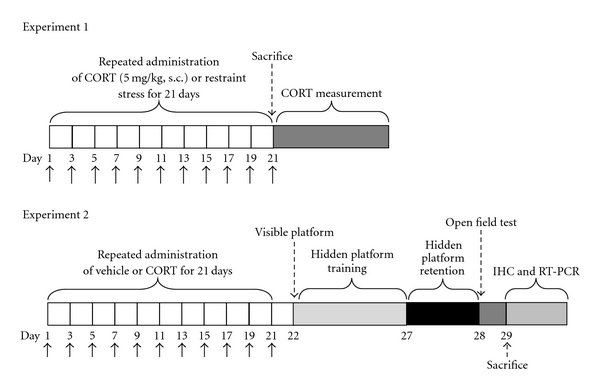
Experimental schedules of corticosterone-induced impairment of spatial memory in the rat. Black arrows indicate acupuncture treatments. Experiment 1 was designed to compare the CORT concentration in rat blood and brain tissue, immediately after CORT injection or immobilization stress for 21 days. It also was designed to compare the effects of acupuncture stimulation to enhanced CORT concentration in the blood of chronic CORT-injected rats for 21 days. Experiment 2 was designed to explore the efficacy of acupuncture therapy for healing chronic CORT-induced spatial memory impairment in an animal model using behavioral and neurobiological methodologies. IHC; Immunohistochemistry.

The experimental schedule of CORT injection, acupuncture treatment, behavioral test, and tissue and blood sampling are shown in Figure 1.

### 2.3. Acupuncture Stimulation

Acupuncture stimulation was bilaterally performed every second day for 5 min before the CORT injection during the CORT-injection period. The acupoint on tail and the Waiguan acupoint were selected as a nonacupoint and a comparison acupoint, respectively. The acupuncture stimulation was performed as previously described [5]. Briefly, small holders with five holes for four limbs and a tail were manufactured to restrain the rat bodies for acupuncturing. During the acupuncture treatment, animals were maintained within the cage with right and left forepaw taken out and fastened to the wall of the cage with tape. Sterilized disposable stainless steel acupuncture needles (0.30 × 25 mm, Suzhou Kangnian Medical Devices Co., Ltd., Shzhou, China) were inserted perpendicularly as deep as 2-3 mm at HT7 or TE5 acupoint. The depth of needle insertion at each acupoint was arbitrarily determined on the basis of several previously studies [20] and in the animal acupuncture atlas [21]. The HT7 point is anatomically located on transverse crease of wrist of forepaw, radial to the tendon of the m. flexor carpi ulnaris. The TE5 point is located 2 inches up to wrist bracelet, 0.5~1.0 inch deep. This acupoint is located between two bones. In addition, the nonacupoint needling was performed at one-fifth point of tail length from the proximal end of the tail to avoid the tail acupoints located at proximal or distal region of the tail. The CON group and CORT group were handled for 5 min before the CORT injection during the CORT-injection period to reduce stress and facilitate handling, instead of acupuncture stimulation, as described previously [5].

### 2.4. Corticosterone Measurement

After immobilization stress or CORT injection for 21 days, CORT concentration in blood and brain tissues was determined. For this, the unanesthetized rats were rapidly decapitated, and blood was quickly collected via the abdominal aorta. The hippocampus was rapidly removed from the rat brains in randomized order. Special care was taken to avoid predecapitation stress—while rats were rapidly decapitated, the other animals were left outside the room and handled for a few minutes prior to sampling. The blood and tissue samples were stored at −80°C until use. Hippocampus were homogenized in a lysis buffer containing 137 mM NaCl, 20 mM Tris (pH 8.0), 1% NP40, 10% glycerol, 1 mM PMSF, 10 mg/mL aprotinin, 1 mg/mL leupeptin and 0.5 mM sodium vanadate. Homogenization was carried out on ice using a tissue homogenizer and incubated for 1 min at 4°C with shaking. Homogenates were centrifuged and supernatants were collected. Protein concentrations were estimated by the procedure of Lowry et al. [22] with BSA as the standard. The CORT concentration was measured by a competitive enzyme-linked immunoassay (ELISA) using a rabbit polyclonal CORT antibody (OCTESIA Corticosterone kit; Alpco Diagnostics Co., Windham, NH, USA) according to the manufacturer's protocol. Samples (or standard) and conjugate were added to each well, and the plate was incubated for 1 h at room temperature without blocking. After wells were washed several times with buffers and proper color developed, the optical density was measured at 450 nm using an ELISA reader (MultiRead 400; Authos Co., Vienna, Austria).

### 2.5. Morris Water Maze Test

#### 2.5.1. Morris Water Maze Apparatus

The MWM test was performed using a polypropylene circular pool (painted white internally, 2.0 m in diameter and 0.35 m high). The pool contained water maintained at a temperature of 22 ± 2°C. The water was made opaque by adding 1 kg of skim milk powder. During the MWM, a platform 15 cm in diameter was located 1.5 cm below the water in one of four sections of the pool, approximately 50 cm from the sidewalls. The pool was surrounded by many cues external to the maze. The pool was divided into four quadrants of equal area. A digital camera was mounted to the ceiling above the pool and was connected to a computerized recording system equipped with a tracking program (S-MART: PanLab Co., Barcelona, Spain), which permitted on- and offline automated tracking of the paths taken by the rat.

#### 2.5.2. Hidden Platform Trial for Acquisition Test

The MWM task was performed on 22st day after the CORT injection and acupuncture treatment were commenced. The animals received three trials per day. The rats were trained to find the hidden platform, which remained in a fixed location throughout the test. The trials lasted for a maximum of 180 s, and the time it took to find the submerged platform was recorded each time. The animals were tested in this way three per day for 6 consecutive days, and they received a 60-s probe trial on the seventh day. Finding the platform was defined as staying on it for at least 4 s before the acquisition time of 180 s ended. If the rat failed to find the platform in the allotted time, it was placed onto the platform for 20 s and assigned a latency of 180 s. Between one trail and the next, water was stirred to erase olfactory traces of previous swim patterns. The entire procedure took seven consecutive days, and each animal had three training trials per day, with a 30- to 40-min intertrial interval.

#### 2.5.3. Visible Platform (Cued Trial) Test

The cued trial (three trials per rat) was performed on the first day (on 22st day) to assess the rats' motivation to escape from the water and to evaluate their sensor-motor integrity. The platform was placed in the north quadrant and had a visible black cue. The animal was placed in the pool and given 90 s to reach the platform, which was identified by a visible black platform above the surface of the water. Latency to reach the visible platform was automatically calculated.

#### 2.5.4. Probe Trial for Retention Test

For the probe trial, each rat was placed into the water diagonally from the target quadrant (north), and for 60 s, was allowed to search the water, from which the platform had been removed. The time (% for total time) spent searching for the platform in the former platform quadrant (north) and in the other three quadrants was measured for each rat.

### 2.6. Open Field Test

Prior to water maze testing, the rats were individually housed in a rectangular container made of dark polyethylene (45 × 45 × 35 cm) in a dimly lit room equipped with a video camera above the center of the floor, and locomotor activity was measured. The locomotor activity was monitored by a computerized video-tracking system using S-MART program (PanLab Co.). The distance they traveled in the container was recorded during the 5-min test. The locomotor activity was measured in meters. The floor surface of each chamber was thoroughly cleaned with 70% ethanol between tests. The Plexiglas square arena was divided into nine equal-sized squares on the floor. The number of lines crossed (with all four paws) between the squares area was recorded for 5 min.

### 2.7. Choline Acetyltransferase (ChAT) Immunohistochemistry

For immunohistochemical studies, four rats in each groups were deeply anesthetized with sodium pentobarbital (80 mg/kg, by intraperitoneal injection) and perfused through the ascending aorta with normal saline (0.9%) followed by 300 mL (per rat) of 4% paraformaldehyde in 0.1 M phosphate-buffered saline (PBS). The brains were removed, postfixed overnight, and cryoprotected with 20% sucrose in 0.1 M PBS at 4°C. Coronal sections 30 *μ*m thick were cut through the septal region or hippocampus using a cryostat (Leica CM1850; Leica Microsystems Ltd., Nussloch, Germany). The sections were obtained according to the rat atlas of Paxinos and Watson (hippocampus; between bregma −2.6 and −3.6) [23]. The sections were immunostained for ChAT expression using the avidin-biotin-peroxidase complex (ABC) method. Briefly, the sections were rinsed three times for 5 min each in PBS and then incubated with primary rabbit anti-ChAT antibody (1 : 2000 dilution; Cambridge Research Biochemicals Co., Bellingham, UK) in PBST (PBS plus 0.3% Triton X-100) for 72 h at 4°C. The sections were washed for 5 min in PBS and then incubated for 120 min at room temperature with biotinylated antirabbit goat IgG (for the anti-ChAT antibody). The secondary antibodies were obtained from Vector Laboratories Co. (Burlingame, Calif, USA) and diluted 1 : 200 in PBST containing 2% normal goat serum. To visualize immunoreactivity, the sections were incubated for 90 min in ABC reagent (Vectastain Elite ABC kit; Vector Labs. Co.), washed three times for 5 min in PBS, and incubated in a solution containing 3,3′-diaminobenzidine (DAB; Sigma) and 0.01% H_2_O_2_ for 1 min. Finally, the tissues were washed in PBS, followed by a brief rinse in distilled water, and mounted individually onto slides. Slides were allowed to air dry and were then cover-slipped. Images were captured using the AxioVision 3.0 imaging system (Carl Zeiss, Inc., Oberkochen, Germany) and processed using Adobe Photoshop (Adobe Systems, Inc., San Jose, Calif, USA). The sections were viewed at 100x magnification, and the numbers of cells within 100 × 100-mm^2^ grids were counted by observers blinded to the experimental groups. Medial septum or hippocampal area cells were obtained according to the stereotactic atlas of Paxinos and Watson [23]. The cells were counted in three sections per rat within the hippocampal area.

### 2.8. Acethylcholinesterase (AchE) Immunohistochemistry

For AchE histochemistry, the sections were washed in PBS and incubated in a solution with 25 mg of acetylthiocholine iodine for 1 h. The solution was composed of 32.5 mL of 0.1 M sodium hydrogen phosphate buffer (NaH_2_PO_4_·H_2_O, pH 6.0), 2.5 mL of 0.1 M sodium citrate, 5 mL of 30 mM copper sulfate, 5 mL of 5 mM potassium ferricyanide, and 5 mL of distilled water. The color of the mixing solution was green. The densities of stained nuclei of the hippocampal cells were measured using a Scion image program (Scion Co., Frederick, Md, USA). The sections were viewed at 200x  magnification, and the numbers of cells within 100 × 100-mm^2^ grids were counted by observers blinded to the experimental groups. Hippocampal area cells were obtained according to the stereotactic atlas of Paxinos and Watson [23]. The cells were counted in three sections per rat within the hippocampal area.

### 2.9. Total RNA Preparation and RT-PCR Analysis

The hippocampus from three rats in each group was isolated. After decapitation, the brain was quickly removed and stored at −80°C until use. Total RNA was isolated from the brain samples using TRIzol reagent (Invitrogen Co., Carlsbad, Calif, USA) and was used to extract RNA according to the supplier's instruction. Complementary DNA was synthesized from total RNA with reverse transcriptase (Takara Co., Shiga, Japan). Brain-derived neurotrophic factor (BDNF) and cAMP-response element-binding protein (CREB) mRNA expression levels were determined by the reverse transcription-polymerase chain reaction (RT-PCR). RT-PCR was performed using a PTC-100 programmable thermal controller (MJ Research, Inc., Watertown, Mass, USA). The operating conditions were as follows: for glyceraldehydes-3-phosphate dehydrogenase (GAPDH), 30 cycles of denaturation at 95°C for 30 sec, annealing at 58°C for 30 sec, and extension at 72°C for 30 sec; for BDNF, 27 cycles of denaturation at 95°C for 30 sec, annealing at 57°C for 30 sec, and extension at 72°C for 30 sec; for CREB, 27 cycles of denaturation at 95°C for 30 sec, annealing at 51°C for 30 sec, and extension at 72°C for 30 sec. All primers were designed using published mRNA sequences and primer design software (Primer 3; The Whitehead Institute for Biomedical Research, Cambridge, Mass, USA; http://www.genome.wi.mit.edu/), offered through the web site. The following sequences were used: for GAPDH (409 bp), (forward) 5′-ATC CCA TCA CCA TCT TCC AG-3′ and (reverse) 5′-CCT GCT TCA CCA CCT TCT TG-3′; for BDNF (153 bp), (forward) 5′-CAG GGG CAT AGA CAA AAG-3′ and (reverse) 5′-CTT CCC CTT TTA ATG GTC-3′; for CREB (183 bp), (forward) 5′-TAC CCA GGG AGG AGC AAT AC-3′ and (reverse) 5′-GAG GCA GCT TGA ACA ACA AC-3′. The PCR products were separated on 1.2% agarose gels and stained with ethidium bromide, and the density of each band was analyzed using an image-analyzing system (i-Max, CoreBio System Co., Seoul, Korea). Complementary DNA expression levels were determined by calculating the relative density of each BDNF or CREB band to GAPDH.

### 2.10. Statistical Analysis

All measurements were performed by an independent investigator blinded to the experimental conditions. Results in figures are expressed as mean ± standard error of means (SE). Differences within or between normally distributed data were analyzed by analysis of variance (ANOVA) using SPSS (Version 13.0; SPSS, Inc., Chicago, Ill, USA) followed by Tukey's* post hoc *test. Statistical significance was set at *P* < 0.05.

 For statistical analysis of water maze data, the effect of training on the acquisition of the water escape task was assessed using a one-way ANOVA with a repeated-measure factor of sessions (number of days) followed by the appropriate Tukey's* post hoc* analysis. For the probe trial in the water maze, within-group differences in the time spent in each quadrant and immunohistochemical data and PCR analysis were also analyzed by one-way ANOVA followed by Tukey's *post hoc* test.

## 3. Results

### 3.1. Experiment 1

#### 3.1.1. Corticosterone (CORT) in the Blood and Brain Tissues

**Figure 2:**
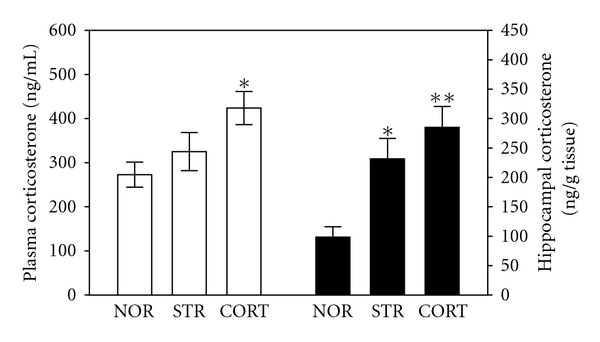
Corticosterone (CORT) concentrations in rat plasma and hippocampus, immediately after CORT injection or immobilization stress for 21 days. The experimental groups were pretreated with nontreated normal (NOR, *n* = 4), restraint-stressed (STR, *n* = 4) and CORT-injected (CORT, *n* = 4) rats. Data were analyzed using a one-way ANOVA followed by Tukey's *post hoc *test. *P < 0.05, **P < 0.01  *versus* NOR group. Vertical bars indicate SE.

The ELISA analysis demonstrated that restraint-stress exposure for 21 days and CORT administration for 21 days significantly increased the plasma CORT concentration in the rats by 19.2% (*P* = 0.593) and 55.4% (*P* < 0.05), respectively, compared with rats in the nontreated NOR group [*F*(2,11) = 4.324; *P* < 0.05]. Additionally, exposure to both restraint stress and exogenous CORT administration induced significant increases in the hippocampal CORT concentration by 135.64% (*P* < 0.05) and 190.2% (*P* < 0.01), respectively, compared with the nontreated NOR group [*F*(2,11) = 9.951; *P* < 0.01] (Figure 2). The CORT concentration of the blood and the hippocampus in the CORT group was higher than that in the nontreated NOR group and was closely associated with that in the STR group. There was no significant difference in CORT concentration between the CORT induction by psychological stress (STR group) and the physiological CORT injection (CORT group). In these results, exogenous CORT-induced memory impairment was exploited to develop a chronic stress model in the rats.

**Figure 3:**
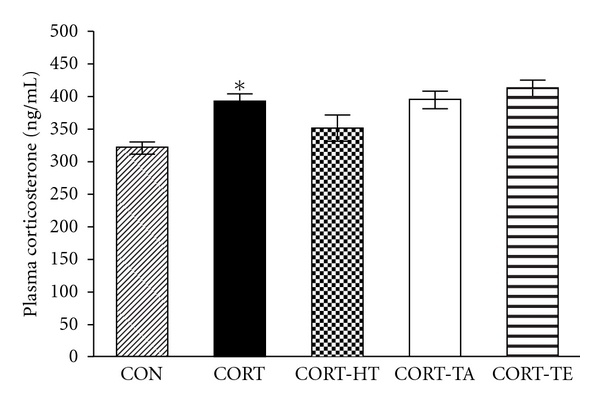
Effect of acupuncture on plasma CORT concentrations induced by repeated CORT injection in the rats. The experimental groups were pretreated with vehicle saline-injected group, instead of CORT (0.9% NaCl, s.c., CON group, *n* = 4), CORT-injected and nontreated group (5 mg/kg, s.c., CORT group; *n* = 4), CORT-injected and Sinmun (HT7) acupoint-stimulated group (CORT-HT group; *n* = 4), CORT-injected and Waiguan (TE5) acupoint-stimulated group (CORT-TE group; *n* = 4), and CORT-injected and nonacupoint- (on the tail) stimulated group (CORT-TA group; *n* = 4). Data were analyzed using a one-way ANOVA followed by Tukey's *post hoc *test. *P < 0.05 *versus* CON group. Vertical bars indicate SE.

 Also, CORT concentration differed among the five groups after acupuncture stimulation [*F*(4,19) = 7.791; *P* < 0.01]. The plasma levels of CORT in the CORT-HT groups lower than that in the CORT group (*P* = 0.190), while the CORT group had significantly higher CORT levels compared to the CON group (*P* < 0.05) (Figure 3).

### 3.2. Experiment 2

#### 3.2.1. Effect of Acupuncture in the Visible-Platform Trial of the Morris Water Maze Test

**Figure 4:**
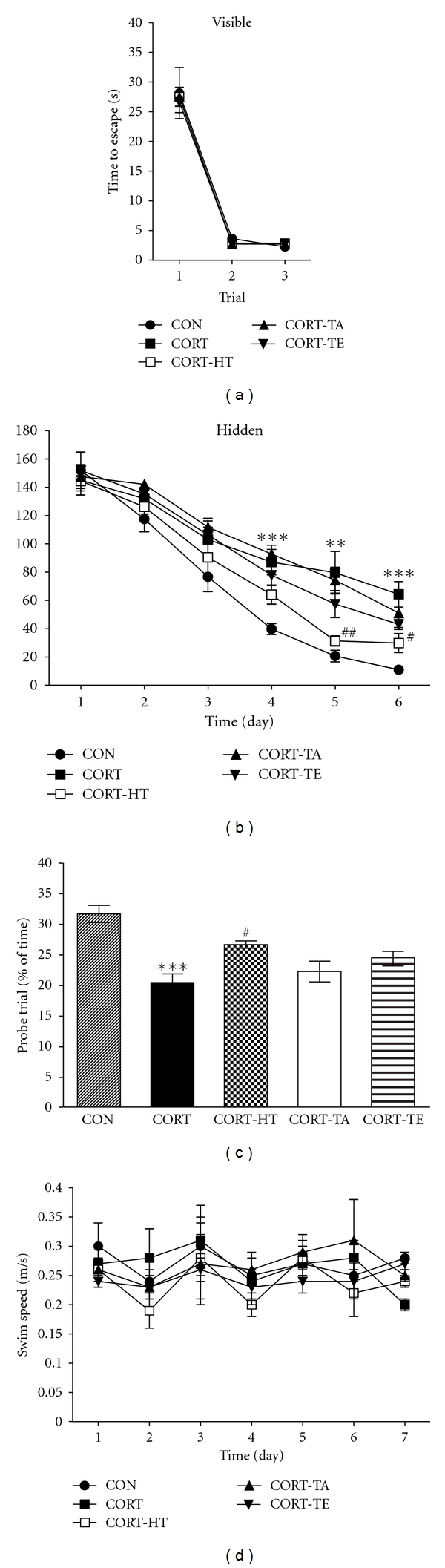
Time to escape (latency) during acquisition trials of visible platform (a), hidden platform (b), probe trial (c), and swim speed (d), during the Morris water maze test. The experimental group were pretreated with vehicle saline, instead of CORT (0.9% NaCl, s.c., CON group, *n* = 7) once daily for 21 consecutive days. The control group were pretreated with CORT (5 mg/kg, s.c., CORT group; *n* = 7) once daily for 21 consecutive days. The other groups were pretreated with CORT-injected and Sinmun (HT7) acupoint-stimulated group (CORT-HT group; *n* = 7), CORT-injected and Waiguan (TE5) acupoint-stimulated group (CORT-TE group; *n* = 7), and CORT-injected and nonacupoint (on the tail)-stimulated group (CORT-TA group; *n* = 7) every second day for 5 min before the CORT injection, respectively. Data were analyzed using a repeated-measures ANOVA followed by Tukey's *post hoc *test. **P < 0.01 and ***P < 0.001  *versus* the CON group; ^#^ *P* < 0.05 and ^##^ *P* < 0.01  *versus *the CORT group. Vertical bars indicate SE.

To exclude the possibility of impairing the animals' vision and changing the motivation to escape the water due to the acupuncture stimulation, a cued version of the MWM test was performed, and the swimming time to reach the visible platform was measured as illustrated in Figure 4(a). When trained to a visible platform, there were no significant differences between groups [*F*(4,30) = 0.116; *P* = 0.976]. As shown in Figure 4(a), the latency to find the visible platform did not differ among groups on the first, second, or third trials. On the second and third trials, the latency was markedly reduced in all groups compared with the first trial. All rats, irrespective of grouping, were able to locate the visible platform more rapidly as the trial number increased. 

#### 3.2.2. Effect of Acupuncture in the Hidden-Platform Trail of the Morris Water Maze Test

The rats in the CON group rapidly learned the location of the submerged hidden platform and reached it within 20 s on day 6 of the trials (Figure 4(b)). The acupoint-stimulated groups also showed a reduction in escape latency throughout the training period, and the CORT group showed a marked retardation in escape latency reduction due to CORT-induced impairment of learning and memory. Analysis of the training data by repeated-measures ANOVA showed that escape latency differed significantly among the groups when the times were averaged over all sessions [*F*(4,30) = 13.745; *P* < 0.001]. During the experiment, escape latency decreased over time [*F*(5,150) = 143.875; *P* < 0.001]. Additionally, a significant interaction between experimental groups and time [*F*(20,150) = 1.788; *P* < 0.05]. Tukey's post hoc test revealed that rats in the CORT-HT group had significantly reduced swimming latency compared with those in the CORT group (*P* < 0.01 on day 5 and *P* < 0.05 on day 6; Figure 4(b)). The CORT group was not significantly different from other groups in terms of mean swimming speed, calculated by dividing the total swim distance by the latency [*F*(4,30) = 0.936; *P* = 0.457] (Figure 4(d)). Total distance traveled in each group was closely associated with escape latency in this task (data not shown). On the basis of these results, the HT7-acupoint-stimulated rats showed improved acquisition in the hidden-platform trial, reaching the platform with lower latency than the CORT-injected and/or nonacupoint-stimulated rats.

#### 3.2.3. Effect of Acupuncture in the Probe Trial of the Morris Water Maze Test

To examine the spatial memory of rats, we analyzed their performance in the probe test on day 7 by comparing the percentage of time spent swimming to the expected position of the platform (Figure 4(c)). The time spent swimming around was significantly reduced in the rats that swam to the target area where the platform had been located [*F*(4,34) = 10.765; *P* < 0.001]. The chronic administration of CORT severely impaired spatial performance in the MWM (*P* < 0.001). Rats in the CORT-HT group spent more time around the platform area than did those in the CORT group (*P* < 0.05). The stimulation of HT7 acupoint significantly attenuated the CORT-induced deficit in learning and memory demonstrated in the water maze task. This study also indicated that the swimming latency in the rats receiving acupuncture stimulation to the acupoint HT7 was higher than that in the rats receiving stimulation to a nonacupoint (CORT-TA) and another acupoint (CORT-TE) as controls. No difference in swimming latency time was observed between the CORT-TA (*P* = 0.881) or CORT-TE groups (*P* = 0.247) compared with the CORT group. Therefore, the HT7-acupoint-stimulated rats also showed a significant amelioration in the memory retention test: they spent more time in the quadrant where the platform was formerly located and swam over the former location of the platform more frequently.

#### 3.2.4. Effect of Acupuncture in the Open-Field Test

**Figure 5:**
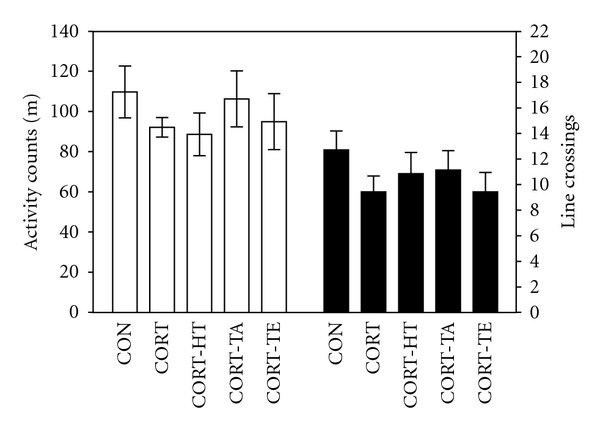
Activity counts of locomotor activity (a) and the total number of line crossings (b) in the open-field test.

In an analysis of open-field test results by a parametric one-way ANOVA, no significant differences were observed between groups in terms of memory deficit-related locomotor activity or the total number of line crossings in the open-field test (Figure 5). The statistical differences in observed locomotor activity and the total number of line crossings between groups were *F*(4,34) = 0.794; *P* = 0.539 and  *F*(4,34) = 1.220; *P* = 0.323, respectively. This indicates that acupuncture stimulation to HT7 acupoint did not affect psychomotor function as measured by the rats' performance in the MWM test.

#### 3.2.5. Effect of Acupuncture on Septal-Hippocampal Choline Acetyltransferase

**Figure 6:**
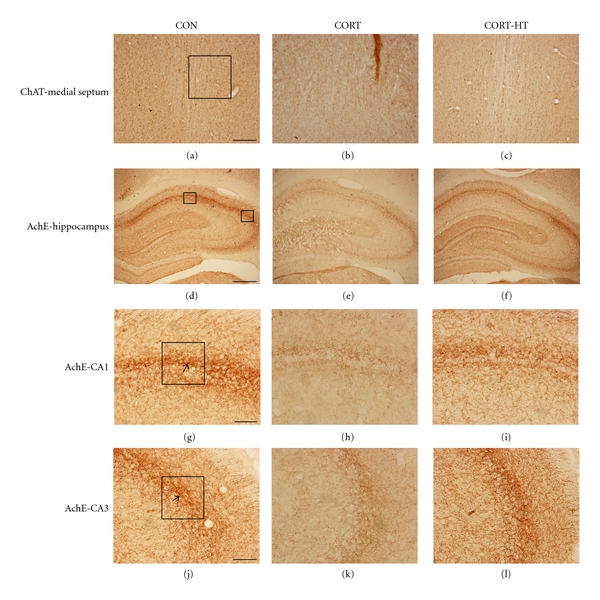
Representative photographs showing the distribution of choline acetyltransferase (ChAT) immunostaining cells in medial septum and acetylcholinesterase (AchE) reactive cells in hippocampus of CORT-induced memory impairment or CORT-injected and Sinmun (HT7) acupoint-stimulated rats. Lower magnification of the small box in panel (d) and high magnification of the big box in panel (g and j) of the same fields of AchE- stained nuclei of the hippocampus. Scale bar represents 100 *μ*m (a), 200 *μ*m (d) and 50 *μ*m (g and j), respectively.

**Figure 7:**
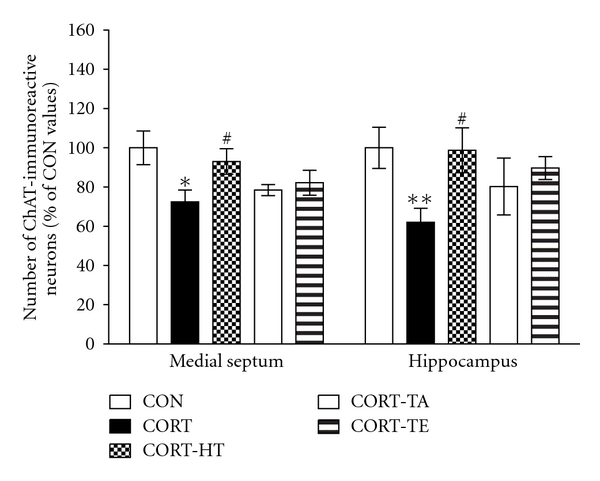
The percentage (±SE) values of the mean number of choline acetyltransferase- (ChAT-) stained septohippocampal cholinergic neurons after the Morris water maze task. Immunohistochemical data were analyzed via a separate one-way ANOVA followed by Tukey's *post hoc* test. **P < 0.01  *versus* the CON group; ^#^ *P* < 0.05  *versus *the CORT group. Vertical bars indicate SE.

The possibility that the deficits in septal-hippocampus-dependent learning and memory of the chronic exposure to CORT are associated with cholinergic deficits was examined histologically following completion of the behavioral tasks using ChAT immunohistochemistry. The number of septal-hippocampal cholinergic neurons immunohistochemically stained for ChAT was counted in the septohippocampal fibers, represented by medial septum or hippocampus. The results of number of ChAT-stained septohippocampal cholinergic neurons are shown in (Figure 6). The brains of the CORT group showed significant decreased ChAT positive cells in the medial seputm compared with the CON group (*P* < 0.05). Comparison of the numbers of ChAT-immunoreactive neurons by a one-way ANOVA revealed a significant differences among groups [*F*(4,79) = 2.869; *P* < 0.05] (Figure 7). The number of ChAT-immunoreactive neurons in the medial septum area was 55.19 ± 3.58 (100.0 ± 10.53%) in the CON group, 34.25 ± 3.92 (62.06 ± 7.11%) in the CORT group, 54.50 ± 6.33 (98.75 ± 11.47%) in the CORT-HT group, 44.31 ± 7.99 (80.29 ± 14.48%) in the CORT-TA group, and 48.88 ± 3.18 (89.68 ± 5.83%) in the CORT-TE group. The number of ChAT-immunoreactive neurons was significantly increased in the medial septum region in the CORT-HT group (*P* < 0.05), compared with the CORT group (Figure 7). The brains of the CORT group showed significant decreased ChAT positive cells in the hippocampal CA1 area, one of the target areas of septohippocampal cholinergic neurons, compared with the CON group (*P* < 0.01). Comparison of the numbers of ChAT-immunoreactive neurons by a one-way ANOVA revealed a significant difference among groups [*F*(4,79) = 4.893; *P* < 0.01] (Figure 7). The number of ChAT-immunoreactive neurons in the hippocampal CA1 area was 92.63 ± 7.40 (100.0 ± 8.56%) in the CON group, 67.13 ± 5.62 (72.47 ± 6.06%) in the CORT group, 86.19 ± 6.00 (93.05 ± 6.48%) in the CORT-HT group, 72.69 ± 2.59 (78.48 ± 2.80%) in the CORT-TA group, and 70.88 ± 5.47 (82.23 ± 6.35%) in the CORT-TE group. The number of ChAT-immunoreactive neurons was significantly increased in the hippocampal CA1 region in the CORT-HT group (*P* < 0.05), compared with the CORT group (Figure 7). The ChAT-immunoreactivity loss in the CORT group was remarkably restored by the HT7 acupoint stimulation, and the ChAT-immunopositive neuron numbers were closely similar to those in the CON group. 

#### 3.2.6. Effect of Acupuncture on Hippocampal Acetylcholinesterase

**Figure 8:**
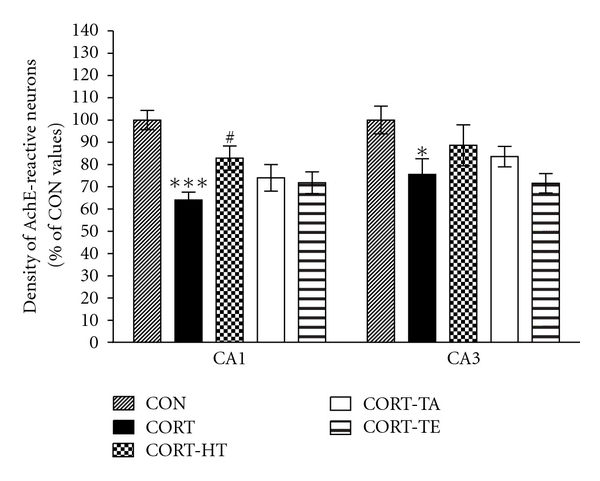
The percentage (±SE) values of the immune-staining density of acetylcholinesterase in different hippocampal areas after the Morris water maze task. Immunohistochemical data were analyzed via a separate one-way ANOVA followed by Tukey's *post hoc* test. *P < 0.05 and ***P < 0.001 *versus* the CON group; ^#^ *P* < 0.05  *versus *the CORT group. Vertical bars indicate SE.

The density of AchE-immunopositive fibers in the CA1 area of the rat hippocampus was significantly reduced by repeated injections of CORT in the CORT group compared with the saline-injected vehicle group (CON group) (Figure 8). The AchE-positive neuron density in the CA1 was 11.31 ± 0.80 (100.0 ± 4.34%) in the CON group, 7.25 ± 0.40 (64.09 ± 3.56%) in the CORT group, 9.38 ± 0.62 (82.87 ± 5.47%) in the CORT-HT group, 8.38 ± 0.68 (74.03 ± 5.98%) in the CORT-TA group, and 8.13 ± 0.55 (71.82 ± 4.90%) in the CORT-TE group [*F*(4,79) = 9.205; *P* < 0.001]. The AchE-reactive neuron loss in the hippocampal CA1 area due to chronic exposure to exogenous CORT was significantly restored by the HT7 acupoint stimulation (*P* < 0.05). The density of AchE-reactive neuron in the HT7 acupoint-stimulated group (CORT-HT group) was closely similar to that in the CON group.

 The density of AchE fiber in the CA3 region of the hippocampus was also markedly reduced by repeated injections of CORT in the CORT group compared with the CON group (Figure 8). The AchE neuron density in the CA3 region was 11.00 ± 0.79 (100.0 ± 6.23%) in the CON group, 8.31 ± 0.78 (75.57 ± 7.08%) in the CORT group, 9.75 ± 1.01 (88.64 ± 9.22%) in the CORT-HT group, 9.19 ± 0.50 (83.52 ± 4.56%) in the CORT-TA group, and 7.88 ± 0.48 (71.59 ± 4.38%) in the CORT-TE group [*F*(4,79) = 3.588; *P* < 0.05]. Tukey's post hoc test showed that the HT7 acupoint stimulation restored the loss of AchE reactive neurons in the CA3 area of the hippocampus, although the change was not statistically significant. The exogenous CORT-induced decreases in AchE-immunoreactive neuron densities were not significantly recovered in the CORT-HT group (*P* = 0.526) compared with the CORT group.

#### 3.2.7. Effect of Acupuncture on BDNF and CREB mRNA Expression in the Hippocampus

**Figure 9:**
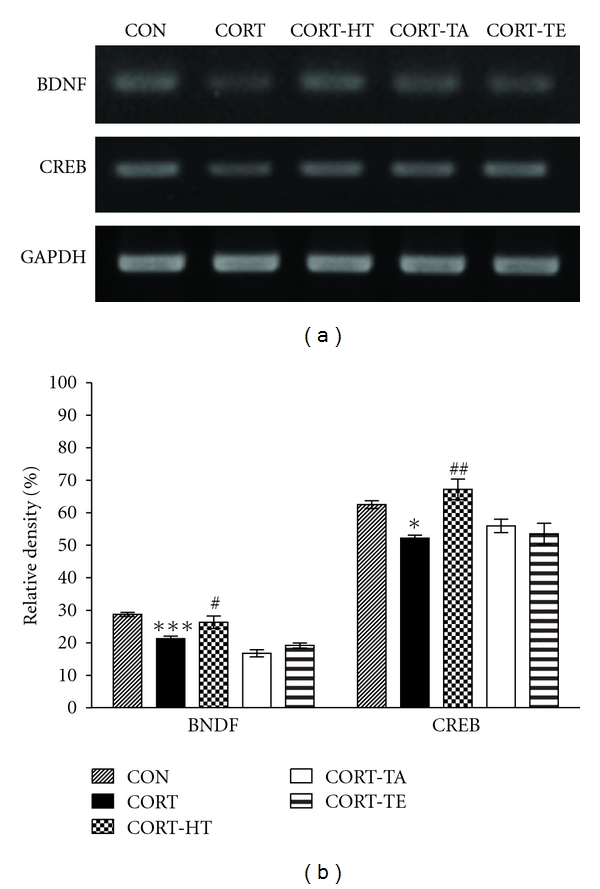
The PCR bands and their relative intensities for brain-derived neurotrophic factor (BDNF) and cAMP-response element-binding (CREB) protein in the hippocampus of rats that received chronic CORT administration. Data were analyzed via separate one-way ANOVAs followed by Tukey's *post hoc* test. *P < 0.05 and ***P < 0.001 *versus* the CON group; ^#^ *P* < 0.05 and ^##^ *P* < 0.01  *versus *the CORT group. Vertical bars indicate SE.

The effect of acupuncture stimulation to the HT7 acupoint on BDFN and CREB mRNA expression levels in the rats with CORT-induced hippocampus lesions was investigated by RT-PCR analysis (Figure 9). The BDNF and CREB mRNA expression levels were normalized against glyceraldehydes-3-phophate dehydrogenase (GAPDH) mRNA, an internal control. The BDNF mRNA expression in the rat hippocampus in the CORT group was significantly decreased compared to that in the CON group (*P* < 0.001). The reduced expression of BDNF mRNAs in the CORT groups was significantly restored by the HT7 acupoint stimulation in the CORT-HT group (*P* < 0.05), and the restored level was similar to that of normal rats in the CON group [*F*(4,14) = 18.830; *P* < 0.001]. The CREB mRNA expression in the rat hippocampus in the CORT group also decreased compared with that in the CON group (*P* < 0.05). The reduced expression of CREB mRNAs in the CORT group was also significantly restored by the HT7 acupoint stimulation in the CORT-HT group (*P* < 0.01), and the restored level was similar to that of normal rats in the CON group [*F*(4,14) = 7.531; *P* < 0.01].

## 4. Discussion

In the present study, acupuncture stimulation to the HT7 acupoint significantly improved learning and memory retention in the MWM and increased ChAT and AchE immunoreactivities in the hippocampus areas of chronic CORT-induced memory impairment male rats. Interestingly, 5-min acupuncture stimulation prior to CORT administration was enough to modulate CORT-induced neurochemical and behavioral responses (data not shown). In addition, only the acupuncture stimulation to the HT7 acupoint elicited significant responses, compared with another acupoint on a different meridian, TE5, or to a nonacupoint on the tail. These results indicate that stimulation of the acupuncture point spreads throughout the body at a rapid rate, and its effect is highly point specific, at least for modulating CORT-induced memory impairments. Furthermore, acupuncture to HT7 was capable of attenuating a complex behavioral syndrome and protecting the hippocampus from deficits.

In traditional oriental medicine, the Sinmun (HT7) is a specific acupoint located on the heart channel, which is also called the “spirit gate” to the pathway that is used clinically to treat mental, psychosomatic, and cognitive disorders [21, 24]. As a comparable control acupoint, we also performed the stimulation to another acupoint, the Waiguan (TE5) acupoint, on a different (large intestine) meridian, and the triple-energizer channel, which is known to treat immune depression and pain/neuropathy of the arm [21]. Although many studies have been tried to elucidate the effects of acupuncture on various diseases, study of the effect of acupuncture on exogenous CORT-induced cognitive deficits and their behavioral and neurochemical responses has not been reported previously.

Many studies very well recognized that dysregulation of the HPA axis by chronic stress or elevated levels of circulating CORT produces hyperactivity of sympathetic adrenomedullary system, such as CORT, corticosteroid-binding-globulin, ACTH, norepinephrine (NE), and epinephrine (E) [25, 26]. In this study, memory impairments induced by repeated injections of CORT were exploited to develop a chronic stress model in rats. The chronic administration of high-dose CORT increased plasma and hippocampal CORT concentrations in the rats, in line with chronic stress models [10]. Accordingly, in animal models, forced sustaining of high CORT levels can affect animal cognition by reducing memory capacity under experimental conditions, and this might be closely associated with the progression or exacerbation of chronically stressful conditions in humans [27, 28]. Therefore, our results showed that the chronic administration of high-dose CORT also produced severe deficits in the performance of cognitive-function tests and decreased the ChAT and AchE activities in the hippocampus, implying neurodegeneration in the brain.

In the CORT-treated rats, reduced spatial cognitive abilities were associated with a significantly increase in plasma and cerebral CORT levels as compared to control group. In present study, we found that acupuncture at HT7 decreased the CORT release in plasma after chronic CORT administration. Our findings may help to explain that stimulation at HT7 acupoint may affect the hippocampus to received biochemical and behavioral signals induced by reduced CORT level in plasma. Therefore, acupuncture at HT7 may modulate the dysregulation of HPA axis, which means acupuncture could influence secretion of CORT, thereby normalizing behavioral and neurochemical response. The effects of acupuncture stimulation might possess a relative specificity on acupoints. The correlation observed between brain cholinergic neurons and improved memory abilities were associated with interaction between CORT concentrations and acupuncture in the control of cognitive processes linked to cholinergic neurons. Accordingly, our results suggest that rewarding effect of acupuncture stimulation of HT7 on exogenous CORT-induced cognitive deficits might be the restoration of HPA axis hyperactivity through the decrease of endogenous CORT levels in the CNS, as noted in some studies [29, 30]. Also, cumulative analgesic effects of acupuncture of HT7 by affecting HPA axis activity were probably associated with regulating hypothalamic beta-endorphin [30].

To assess spatial learning and memory in rats, the MWM is more advantageous than other conventional mazes such as the T maze and the radial-arm maze. Training for spatial memory can be easily achieved after several acquisition trials, and the task does not require strong motivating traces or conditions such as scent, punishments, and food and water deprivation. The MWM is a hippocampus-dependent memory task, frequently used for demonstrating cognitive deficits and to examine permanent spatial learning capability and reference memory in rodents [31]. Animals encode spatial working information during the learning step, which serves to guide future memory retrieval. Our findings that the memory deficits, induced by chronic administration of CORT, produced impaired behavioral performance in the MWM are consistent with previous findings [19]. In previous studies, the hidden platform trials were designed mainly to measure acquisition of spatial memory, and the probe trial was used to evaluate retention.

In terms of average swim speed and rest time, which are indices of motor function, the CORT group was not significantly different from HT7-acupuncture group. This indicates that motor impairment was not the main cause of the poor performance of the CORT group in the MWM test. Accordingly, it is evident that acupuncture at the HT7 acupoint significantly improved MWM performance by enhancing spatial working memory.

An open-field test was also performed to rule out any confounding motor impairments, which can influence outcomes in many behavioral tests of depression. No significant individual differences in locomotor activities were observed between groups in the open-field test, suggesting that acupuncture stimulation to the HT7 acupoint had no effect on sensorimotor performance. Accordingly, the changes in behavioral performance in the MWM task were likely due to improved memory instead of differences in sensorimotor function, motor output, or limb flexibility.

Many reports have verified that sustained elevation of circulating GC concentrations produces a variety of cognitive deficits. For instance, rats exposed to daily CORT injections for 8 weeks demonstrated decreased spontaneous alteration in the T maze [18]. Likewise, a 21-day CORT implant that elicited a two- to fourfold increase in serum CORT level impaired acquisition of a passive-avoidance task in rats [32]. In addition, chronic CORT treatment significantly impaired both acquisition in the radial arm maze and accuracy of recall of spatial information in the MWM in rats [19]. In our study, it is likely that poor performance in the MWM task by the rats administered chronic CORT was also attributable to impaired memory [16].

Chronic CORT administration might increase circulating GC levels in the CA1 region of the rat hippocampus, resulting in impairment of long-term potentiation and spatial learning and memory in the MWM [33]. The beneficial effects of acupuncture on CORT-induced learning and memory deficits could be related to an increase in septohippocampal cholinergic function and prevention of degeneration in the cholinergic neuronal population of the septohippocampal fibers in mediating cognitive processes [34]. This effect results in enhanced cognitive performance and abrogation of memory deficit [13]. Several studies have demonstrated the changes in hippocampal neurochemistry in response to chronic administration of exogenous CORT [10, 35]. A prolonged administration of CORT or higher dose of CORT influenced reduction of cholinergic neurons in hippocampus [36]. For example, GC interfered with the storage and inactivation of ACh, via the upregulation of cholinesterase activity [37]. In addition, recent studies have shown that GC receptors on cortical neural stem cells can negatively affected neurogenesis with a subsequent unfavourable functional outcome in the cognitive abilities [38, 39]. Furthermore, deletion adjacent to glucocorticoid-responsive element (GRE) caused constitutive overexpression and anti-AchE hypersensitivity [40], suggesting a physiologically significant role for GC in regulating both neuronal AchE gene expression and anticholinesterase hypersensitivity. Therefore, our results have shown that chronic administration of exogenous CORT induced reduction AchE-stained neurons and ChAT activity in septohippocampal cholinergic neuron, which consistent with those of previous studies [41].

Cholinergic neurons originating in the medial septum (MS) project to the cortex and hippocampus, which play key roles in ACh-associated cognition [42]. Therefore, prolonged administration of CORT reduced spatial cognitive abilities and the number of hippocampal neurons, since the hippocampus received an extensive cholinergic input from the MS area [43]. In the present study, we found that exogenous CORT administration had a reduction in ChAT activity in the MS and hippocampus. The density of AchE-stained neurons in the hippocampal CA1 and CA3 regions was also significantly reduced after chronic CORT administration, which supports the notion of reduced septohippocampal cholinergic neurons. It is relevant to suggest that the reduction in cholinergic neuron loss after acupuncture treatment appears to be associated with improvement of learning and memory, since the CORT-HT group had greater cholinergic markers, such as ChAT and AchE, than the CORT group in the septohippocampal pathway.

The treatment of chronic CORT-induced memory disorders with acupuncture stimulation to the HT7 acupoint might enhances Ach recycling and efficient choline reutilization, and concomitantly induces increases in ChAT and AchE activities. AchE and ChAT belong to a family of enzymatic proteins that are extensively expressed in cholinergic neurons. ChAT is responsible for ACh biosynthesis and is required for cholinergic neurotransmission in the central and peripheral nervous systems. Acetylcholine is rapidly hydrolyzed by AchE; therefore, the duration of ACh action in the synaptic cleft is dependent upon AchE activity [44]. We demonstrated that acupuncture stimulation to the HT7 acupoint protected rats from spatial working memory deficits and attenuated the decrease in AchE and ChAT-immunoreactive neurons in the hippocampus, which is a particularly vulnerable region of the brain.

In the present study, we did not know precisely whether the acupuncture stimulation affects memory improvement through neurochemical changes of cholinergic system or first causes memory improvement leading to the neurochemical changes, because we did not have time-course data of Morris water maze task and cholinergic immunoreactivity in the brain hippocampus. However, CORT levels in the blood were almost restored to the normal level right after the acupuncture stimulation at HT-7 in the CORT-HT group despite little statistical significance (Figure 3). And also time to escape from hidden platform in the CORT-HT group was getting shorter than other CORT-treated groups as the trials were repeated during the hidden platform training period (Figure 4(b)). These observations implied that the acupuncture stimulation at HT-7 first modulated brain cholinergic system and this effect subsequently elicited the behavioral consequences in the Morris water maze task.

In the acupuncture treatment against various memory-related disorders, the salvage capacity of ACh is enhanced. It also improves cholinergic neurons in the frontoparietal cortex and CA1 region of the hippocampus and continuously induces increases in ChAT and AchE activities, which eventually results in recovery of the entire cholinergic circulation pathway [45]. It is likely that the observed improvement in learning and memory in the HT7-acupuncture rats was associated with the attenuation of hippocampal cell loss.

We thus propose that chronic exposure to CORT triggers dysregulation of the HPA axis, which, in turn, elicits a reduction of ChAT and AchE expression in the hippocampus and eventually causes memory and cognitive decline in the rats. Also, recent studies have shown that chronic CORT-induced HPA axis hyperactivity or excessive increase of GC levels regulated expression of BDNF and CREB and impacted function of the BDNF pathway in the hippocampus [46]. This previous study strongly suggests a close correlation between the reduced expression of BDNF and CREB and HPA axis abnormalities in pathogenesis [46].

On the other hand, recent experimental evidence strongly supports the role of hippocampal BDNF in learning and memory processes, besides its actions on neuronal cell survival and prevention of neurodegeneration [47–49]. There is also sufficient evidence that the CREB regulates the expression of genes involved in neuroplasticity, cell survival, and cognition [50]. The phosphorylated CREBs are able to bind to cAMP response elements of the target genes considered to be involved in memory formation [51]. Thus, BDNF transcription, regulated by CREB, may also be a critical player in the adaptive neuronal responses underlying learning and memory function [52]. Several studies have suggested an association of hippocampal BDNF and CREB with memory performance, particularly in the water maze test [53]. The CORT-induced memory deficits led to a significant reduction of BDNF and CREB expression in the hippocampus, as well as poor performance in the learning and memory tests [17].

In oriental traditional medicine, acupuncture improves reversible malfunctions of the body via direct activation of various brain pathways, and thus contributes to the restoration of normal systemic balance, probably due to regulation of neurotransmitters including Ach [54]. Currently, acupuncture is a relevant therapy in complementary and alternative medicine for managing various cognitive disorders and psychosomatic diseases such as stress, depression, and anxiety [5, 55, 56]. Many recent studies also demonstrated that acupuncture stimulation reduced immobilization-stress-induced elevation of CORT levels and modulated HPA axis function in animals [3]. Acupuncture stimulation to the HT7 acupoint had potential effects on brain function in Alzheimer's disease patients, and ST36 (Zusanli) stimulation exerted a protective effect on cognitive impairment caused by cerebral multi-infarct dementia in rats [57]. The stimulation of acupoints such as Zusanli (ST36) and Xuehai (SP10) alleviated memory impairment induced by cerebral multi-infarction, as evaluated by shortened escape latency and increased swimming time in the target quadrant in rats [58]. These findings suggest that acupuncture stimulation can ameliorate memory-related performance in many behavior tests and modulate cholinergic neurons.

## 5. Conclusions

The present study demonstrated that memory and cognitive deficits induced by exogenous CORT-induced septohippocampal cholinergic neuron loss were closely related to the degeneration of cholinergic neurons in the rat hippocampus and that acupuncture stimulation to the HT7 acupoint significantly ameliorated learning and memory deficits through recovery of the ACh system. Acupuncture improved performance on the spatial memory test and protected septohippocampal cholinergic neurons from exogenous CORT-induced destruction. The attenuation of impairments of memory and cognition by acupuncture stimulation might be due to the restoration of cholinergic neurochemical abnormalities. It is most likely that acupuncture therapy is strongly effective in protecting against memory-related neuronal degeneration in the brain and retards the progression of memory defects in neurodegenerative diseases.
